# Ultrafine MnO_2_ Nanowire Arrays Grown on Carbon Fibers for High-Performance Supercapacitors

**DOI:** 10.1186/s11671-016-1693-1

**Published:** 2016-10-20

**Authors:** Jiyu Hu, Feng Qian, Guosheng Song, Wenyao Li, Linlin Wang

**Affiliations:** 1 No.2 High School Attached to East China Normal University, Shanghai, 201203 People’s Republic of China; 2 Institute of Functional Nano & Soft Materials (FUNSOM), Collaborative Innovation Center of Suzhou Nano Science and Technology, Soochow University, Suzhou, Jiangsu People’s Republic of China; 3 School of Material Engineering, Shanghai University of Engineering Science, Shanghai, 201620 People’s Republic of China; 4 College of Chemistry and Chemical Engineering, Shanghai University of Engineering Science, Shanghai, 201620 People’s Republic of China

**Keywords:** CF@MnO_2_, Ultrafine nanowires, Large area, Supercapacitors

## Abstract

Large-area ultrafine MnO_2_ nanowire arrays (NWA) directly grew on a carbon fiber (CF, used as a substrate) by a simple electrochemical method, forming three-dimensional (3D) hierarchical heterostructures of a CF@MnO_2_ NWA composite. As an electrode for supercapacitors, the CF@MnO_2_ NWA composite exhibits excellent electrochemical performances including high specific capacitance (321.3 F g^−1^ at 1000 mA g^−1^) and good rate capability. Further, the overall capacitance retention is ~99.7 % capacitance after 3000 cycles. These outstanding electrochemical performances attribute to a large number of transport channels for the penetration of electrolyte and the transportation of ions and electrons of electrodes. The as-prepared CF@MnO_2_ NWA composite may be a promising electrode material for high-performance supercapacitors.

## Background

For electrochemical energy storage applications, a nanoscale solution to supercapacitors has attracted considerable attention due to their unique advantages such as faster charging/discharging rate, higher power density, much longer lifetimes, and safer operation [1–4]. Up to now, the design and synthesis of nanomaterials have promoted significant advancements in supercapacitors. One-dimensional (1D) nanostructures are believed to facilitate the electrical transport along the axial direction [5]. So far, a wide variety of nanowires, including carbonaceous materials [6, 7], transition metal oxides [8–14], conducting polymers [15, 16], and hybrid composites [17–19], have been synthesized to acquire enhanced electrochemical properties as an electrode in supercapacitors. Among them, 1D nanostructured transition metal oxides with high capacity and low cost have been a popular topic. In particular, manganese oxide (α-MnO_2_) with high theoretical pseudocapacitance (~1370 F g^−1^) has attracted intense attention due to a one-electron transfer and the complete reduction of Mn^IV^ to Mn^III^ as well as its environmental compatibility and earth abundance [20]. However, its poor intrinsically electrical conductivity (10^−5^ to 10^−6^ S cm^−1^) and large volume expansion during repeated cycling processes will limit its practical applications in supercapacitors [21, 22]. Thus, the design and synthesis of an electrode material based on MnO_2_ nanowires that provides a high electrical conductivity and a reduced volume expansion are needed.

Recently, three-dimensional (3D) hierarchical heterostructures by assembling 1D MnO_2_ nanostructures and conductive backbones, e.g., carbon materials [22, 23], nickel foam [24], and Co_3_O_4_ [25], have been demonstrated to show improved electrochemical properties in supercapacitors. In particular, a constitution within 3D heterostructures made of dense ultrafine nanowire arrays will result in high specific surface area and plentiful porosities, forming a great number of electrochemically active sites with shorter diffusion pathways for ions and electrons [26, 27]. Thus, the active materials of the electrode will improve their utilization, i.e., easily participate in reversible redox reactions with the electrolyte solution, enhancing electrochemical kinetics during the charging and discharging process [12–27]. For samples, 3D Co_3_O_4_@MnO_2_ hierarchical nanoneedle arrays by a hydrothermal approach showed excellent electrochemical performances such as high specific capacitances of 932.8 F g^−1^ at a scan rate of 10 mV s^−1^ as well as long-term cycling stability [25]; hierarchical CNTs@NCS@MnO_2_ core-shell composites via a chemical polymerization coating followed by a hydrothermal process exhibited a high specific capacitance of 312.5 F g^−1^ at a current density of 1 A g^−1^ and a good rate capability (76.8 % retention with the charge-discharge rate increasing from 1 to 10 A g^−1^) [28]. However, in the two cases, using a special template (Co_3_O_4_ nanoneedles or CNTs) with unavailable sizes can make large-scale preparation and manipulations more difficult.

Herein, using commercial carbon fibers (CF) as a substrate, large-area ultrafine MnO_2_ nanowire arrays (NWA) directly grew on a CF, forming 3D hierarchical heterostructures of a CF@MnO_2_ NWA composite by a simple electrochemical method. The as-fabricated electrodes by the CF@MnO_2_ NWA composite exhibited an improved specific capacitance of 321.3 F g^−1^ at 1000 mA g^−1^ and an excellent cycling stability in 0.5 M Na_2_SO_4_ aqueous solution, i.e., the specific capacitance of the electrodes showing 99.7 % retention after 3000 cycles.

## Methods

### Synthesis of CF@MnO_2_ NWA Composite

Firstly, a piece of carbon fibers (~4 × 1 cm^2^) was carefully cleaned with deionized water and absolute ethanol in sequence for several times. Secondly, the electrochemical deposition was carried out in a standard three-electrode glass cell consisting of a clean carbon fiber working electrode, a platinum plate (~1.5 × 1.5 cm^2^) counter electrode, and a saturated calomel reference electrode (SCE). MnO_2_ nanowire arrays were electrodeposited on the carbon fibers using an Autolab electrochemical workstation (PGSTAT302N potentiostat), in which deposition conditions included a current density of 0.75 mA cm^−2^, a solution at 70 ± 2 °C containing 0.1 M manganese acetate (Mn(CH_3_COO)_2_) and 0.02 M ammonium acetate (CH_3_CO_2_NH_4_) with 10 % dimethyl sulfoxide (DMSO), and an electrodeposit surface area of 1 × 1 cm^2^. The electrodeposition process was carried out in a water bath, in which the temperature was carefully set at 70 °C. After deposition for 20 min, the CF@MnO_2_ NWA composite was ultrasonically washed with deionized water and absolute ethanol several times and then placed in a vacuum oven at 60 °C for 2 h. Finally, the as-prepared CF@MnO_2_ NWA composite was annealed at 200 °C for 3 h in air. The mass of the CF@MnO_2_ NWA composite was obtained by a weight difference before and after deposition, and the mass of active material MnO_2_ per unit area (1 × 1 cm^2^) of the electrode is about ~1.27 mg.

### Material Characterizations

Powder X-ray diffraction (XRD; Rigaku with Cu-Kα radiation and a normal *θ*–2*θ* scan) was used to characterize the phases in the collected products. Morphological observation and structural analysis of the products were carried out with a scanning electron microscope (SEM; S-4800) and a transmission electron microscopy (TEM; JEM-2100F operated at 200 kV) equipped with an energy-dispersive X-ray spectrometer (EDX). The mass of the electrode materials was weighed on an XS analytical balance (Mettler Toledo; *δ* = 0.01 mg).

### Electrochemical Characterization

Electrochemical performances were performed on the Autolab electrochemical workstation using a three-electrode mode in a 0.5 M Na_2_SO_4_ solution. The reference electrode was a SCE, and the counter electrode was a platinum plate. Standard current-voltage curves were recorded in a potential range of −0.1 to 0.9 V.

## Results and Discussion

**Fig. 1:**
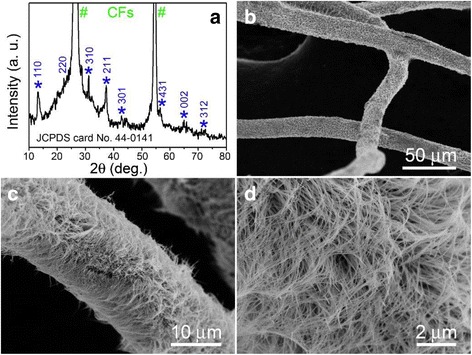
**a** XRD pattern of the as-grown MnO_2_ nanowires on carbon fibers. **b**–**d** Different-magnification SEM images of the CF@MnO_2_ NWA

The phase of the as-grown products was characterized by XRD, as shown in Fig. 1a. In this pattern, two strong peaks marked with “#” are originated from the carbon fibers (substrate) and the other peaks marked with “*” can be indexed to those of the tetragonal phase of α-MnO_2_ (JCPDS: 44-0141). No peaks associated with other crystalline forms of manganese oxide were detected in the pattern. The results indicate a high purity of MnO_2_ material on the carbon fibers. Figure 1b, c shows a low-magnification SEM image revealing the general morphology of the product. It can be seen that the MnO_2_ nanowires grew densely and uniformly on the surface of each carbon fiber, forming a CF@MnO_2_ NWA composite. In fact, these dense MnO_2_ nanowires were intersected with each other to form numerous voids or space clearances, as suggested by a high-magnification SEM image in Fig. 1d. Here, the commercial carbon fibers can also be used as a roll-to-roll media to realize mass production of the CF@MnO_2_ NWA composite, which is expected to continuously fabricate supercapacitors in the future [29].

**Fig. 2:**
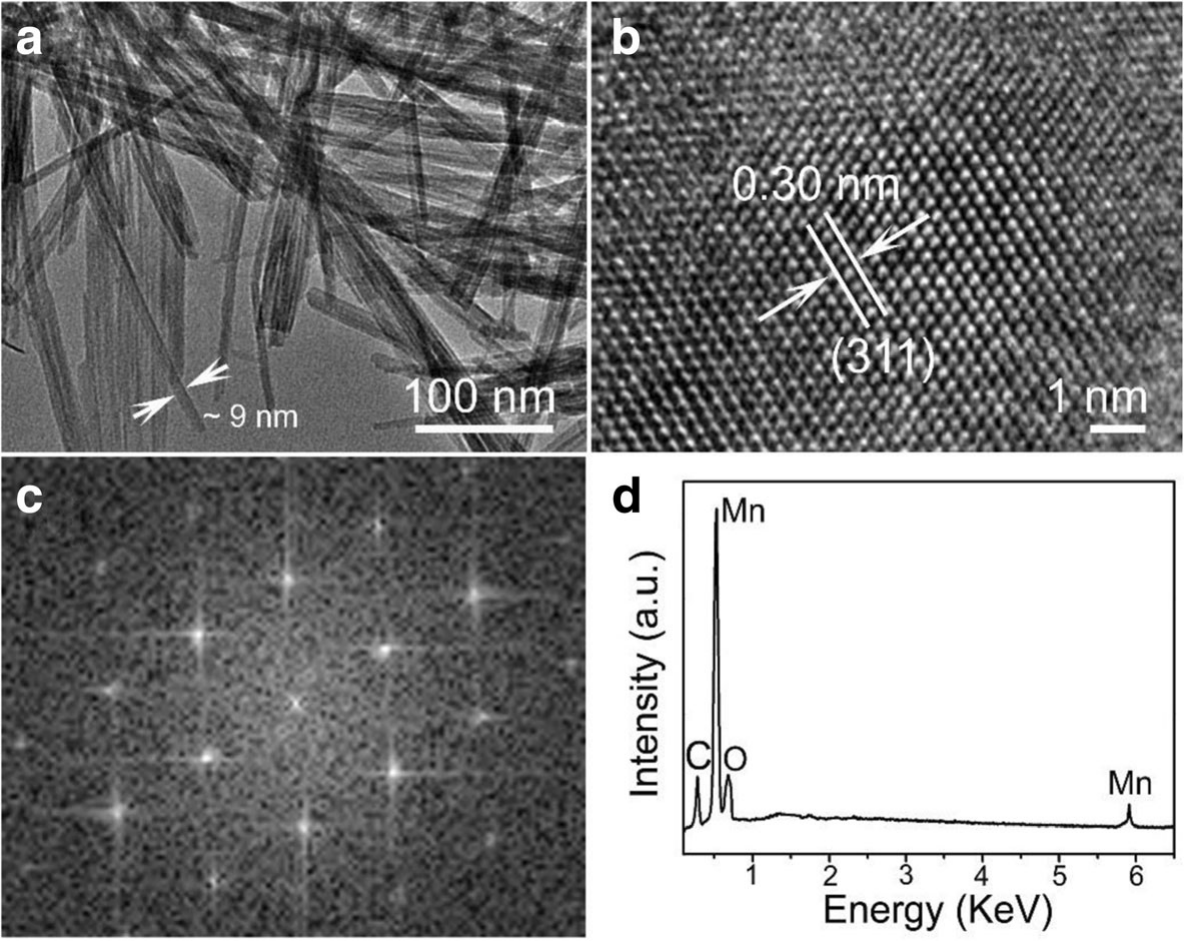
**a** TEM image of the ultrafine MnO_2_ nanowires with a diameter of ~9 nm. **b** HRTEM image of a MnO_2_ nanowire and **c** its corresponding FFT pattern. **d** EDX of the MnO_2_ nanowires

The TEM image and the EDX pattern of the CF@MnO_2_ NWA composite are shown in Fig. 2. Each MnO_2_ nanowire has a length of ~1.5 μm (Fig. 2a). Notably, individual MnO_2_ nanowires have a diameter as fine as ~9 nm. Combining the SEM observation, the as-grown composite made of ultrafine MnO_2_ nanowire arrays and carbon fibers has remarkable advantages for its application as an electrode in the supercapacitors. Clearly, those numerous voids or space clearances among the ultrafine nanowires can provide a large number of transport channels for the penetration of electrolyte and the transportation of ions and electrons of electrodes. Furthermore, the ultrafine MnO_2_ nanowires could greatly increase the specific surface area of the composite. These characteristics will promote the electrochemical reactions to occur on the surface and interface of the composite, finally improving the performance of the supercapacitors. The high-resolution TEM image in Fig. 2b reveals the crystalline nature of the MnO_2_ nanowire, and the observed *d*-spacing of 0.30 nm matches well with the (110) facets of the tetragonal phase of α-MnO_2_. The corresponding fast Fourier transform (FFT) pattern in Fig. 2c agrees with the [110] zone axis of the tetragonal phase of α-MnO_2_. Figure 2d presents the EDX of the MnO_2_ nanowires, which clearly indicates the presence of Mn and O elements (C signals originate from the TEM grid).

**Fig. 3:**
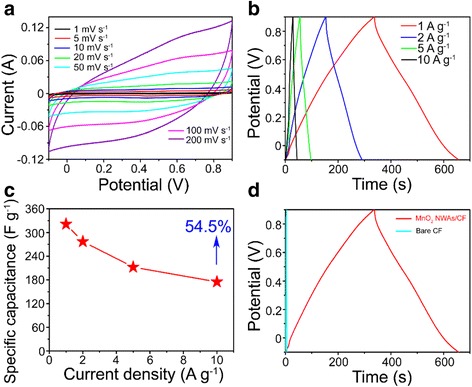
**a** CV curves of the CF@MnO_2_ NWA electrode under different scan rates. **b** Galvanostatic charge-discharge curves of the CF@MnO_2_ NWA electrode at different current densities. **c** Specific capacitance of the CF@MnO_2_ NWA electrode as a function of the current density. **d** Galvanostatic charge-discharge curves of the CF@MnO_2_ NWA and the bare CF at a current density of 1 A g^−1^

Such a composite of the ultrafine MnO_2_ nanowire arrays directly grown on carbon fibers is used as a bind-free electrode for the supercapacitor which was examined in a 0.5 M Na_2_SO_4_ solution. Figure 3a gives cyclic voltammetry (CV) curves of the as-fabricated CF@MnO_2_ NWA electrode at different scan rates. All the curves display a nearly rectangular and symmetric characteristic, which suggests an ideal pseudocapacitive nature with fast charge and discharge processes. This could be thanks to the electrolyte coming in great numbers to the active surface of the electrode materials. Galvanostatic charge-discharge curves of the CF@MnO_2_ NWA electrode were recorded between a potential window range of −0.1 to 0.9 V at various current densities, as shown in Fig. 3b. They appear to be an almost symmetric shape, which reflects an ideal electrochemical capacitive nature and good reversible redox reaction in the whole potential range.

Based on the discharge curves, the discharge-specific capacitance can be obtained referring to the following formula [30]:

1 $$ C = I\cdotp \Delta t/\left(\Delta V\cdotp m\right) $$

where *C* is the specific capacitance (F g^−1^) and *I*, Δ*t*, Δ*V*, and *m* are the discharge current (A), the discharge time (s) taken in the potential range, the potential windows (V), and the mass of the active materials (or the mass of the total electrode materials) (g), respectively. In line with the formula, the discharge-specific capacitances of the electrode material (CF@MnO_2_ NWA) were calculated from the discharge curves to be 321.3, 277, 212.5, and 175 F g^−1^ at the current densities of 1000, 2000, 5000, and 10,000 mA g^−1^, respectively, as shown in Fig. 3c. These specific capacitances here are much higher than that in the reported works on this material with a similar composite structure [31–34].

As we know, for the supercapacitors, the rate capability is also an important aspect in their high-power applications. Figure 3c reflects the specific capacitance of the CF@MnO_2_ NWA electrode measured at different current densities. As can be seen, the CF@MnO_2_ NWA electrode kept 54.5 % of its specific capacitance (from 321.3 to 175 F g^−1^) as the current density increased from 1000 to 10,000 mA g^−1^, i.e., 10 times increase. The excellent rate capability also ascribed to the dense ultrafine space clearances inside the CF@MnO_2_ NWA composite. Obviously, the space clearances can support numerous electrolytic accessible passageways and thus greatly help the electrolyte to penetrate into the active materials, effectively promoting the redox reactions that occurred on the surfaces and interfaces. So, even at a high rate, a considerably high specific capacitance can be obtained. In addition, these numerous passageways within the CF@MnO_2_ NWA composite are expected to slow down the volume expansion upon a long-term cycle of repeating CV test [35]. Noticeably, the CF substrate could also affect the final capacitance of the composite electrode material. By comparing the specific capacitances of the blank CF and the CF@MnO_2_ NWA electrode (Fig. 3d), the influence from the bare CF can be nearly negligible.

**Fig. 4:**
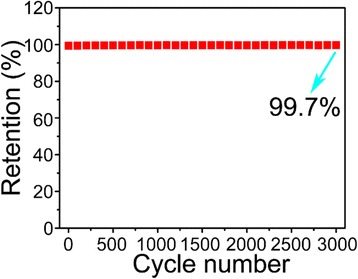
Cycling stability of the CF@MnO_2_ NWA electrode for 3000 cycles at a scan rate of 50 mV s^−1^

A high stability should also be required by high-performance supercapacitors. Here, a long-term cycle stability of an as-fabricated CF@MnO_2_ NWA electrode was evaluated by repeating CV test for 3000 cycles at a scan rate of 50 mV s^−1^. As shown in Fig. 4, after 3000 cycles, the capacitance retention is as high as ~99.7 %, that is, the overall loss of the capacitance is as low as ~0.3 %. In other words, this electrode represented a capacity decay rate per cycle of only 0.0001 %, which is considerably lower than those of the reported MnO_2_-based cathodes [31–33, 36, 37]. The credit for such high stability could be given to those numerous ultrafine spaces inside the MnO_2_ nanowire as well as the good electrical conductivity from the carbon fibers, of which the electron collection became facile and much more electrons became involved in the electrochemical reactions. It is believed that the electrode material of the CF@MnO_2_ NWA composite can be highly competitive for a long cycle life.

## Conclusions

In summary, large-area ultrafine MnO_2_ nanowire arrays were directly grown on carbon fiber substrates resulting in a 3D CF@MnO_2_ NWA composite by an easy electrochemical deposition. The CF@MnO_2_ NWA electrode demonstrates excellent electrochemical performances, i.e., ultrahigh specific capacitances of 321.3 and 175 F g^−1^ at current densities of 1000 and 10,000 mA g^−1^, respectively, a good rate capability, and a long cycling stability with a capacitance loss of 0.3 % after 3000 cycles. These overall fine electrochemical performances should owe to the effective electron and ion-transport pathways that originated from the distinctively microstructural characteristics of the CF@MnO_2_ NWA composite. So, it makes the CF@MnO_2_ NWA composite a promising electrode material for the high-performance supercapacitors.
